# Sex-specific rejection in mate-guarding pair formation in the intertidal copepod, *Tigriopus californicus*

**DOI:** 10.1371/journal.pone.0183758

**Published:** 2017-08-23

**Authors:** Satomi Tsuboko-Ishii, Ronald S. Burton

**Affiliations:** Marine Biology Research Division, Scripps Institution of Oceanography, University of California, San Diego, La Jolla, California, United States of America; University of Missouri Columbia, UNITED STATES

## Abstract

Securing a potential mate is one of the most important processes in sexual reproduction of animals. Intertidal copepods of the genus *Tigriopus* show mate-guarding behavior where a male captures a female and continues to clasp her for up to two weeks prior to copulation. Although these copepods form a mate-guarding pair between a male and a female with high accuracy, interactions between the sexes in pair formation have not been well described and the mechanism allowing successful male-female pair formation is not yet understood. In this study, we performed experiments with *Tigriopus californicus* to analyze the behavior of both a capturer (male) and a captured individual (female or male) in formation of a guarding pair. While capturer males were attracted by both females and males, capture of virgin males was terminated in a significantly shorter time than that of virgin females. However, when presented freshly killed females or males, regardless of the sex of the body, capturer males continued to clasp the body for a comparable time as in an attempt on a living female. Our results suggest that a sex-specific rejection signal actively sent by captured males prevents male-male pair formation. Experiments also suggest that mated females reject an attempt of pair formation. To our knowledge, this is the first study to suggest involvement of active rejection by a captured individual in facilitation of reproductively successful male-female guarding pair formation in the genus *Tigriopus*.

## Introduction

Sexually reproducing animals employ various strategies for securing a mate. In a wide range of animal taxa including crustaceans [1], insects [2], teleosts [3], rodents [4], primates [5], and humans [6], sexually mature individuals perform mate-guarding behavior, where they physically contact or stay proximate to their potential mate to prevent it from copulating with other individuals. To increase reproductive outcome from the investment, guarding individuals often assess qualities of a guarding target (e.g. sex, developmental stage, potential fertility) before and/or in the process of mate guarding [7].

**Fig 1:**
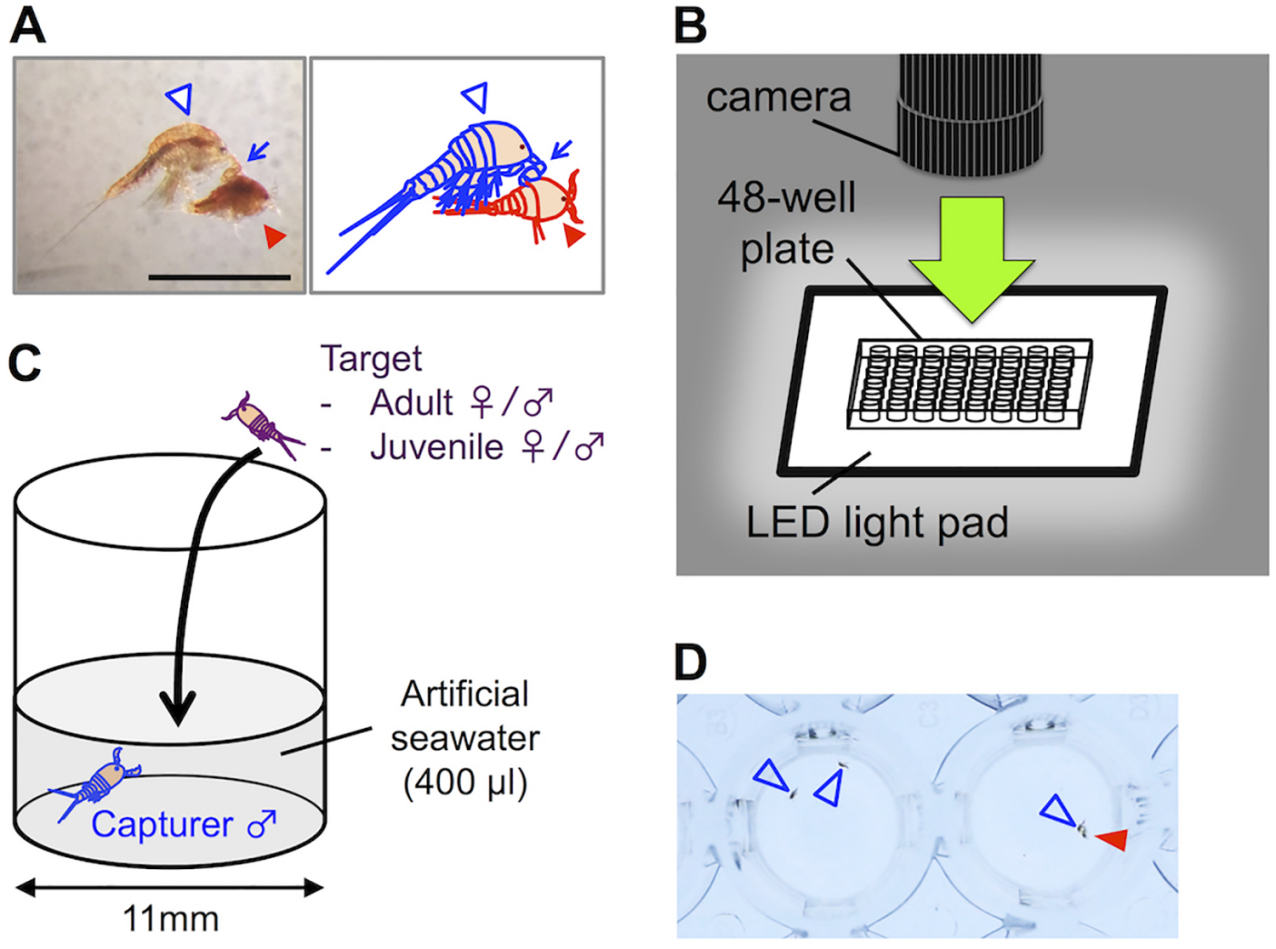
Outlines of mate-guarding behavior and the behavioral tests. (A) An adult male (capturer: indicated by white outlined arrowheads) clasping a juvenile (target: indicated by red arrowheads) with antennae (indicated by blue arrows). Bar = 1 mm. (B) Setup for video recording of guarding attempts. (C) An outline of behavioral tests. (D) Still image from a video recording. Arrowheads were added to indicate tested animals. The left well has unpaired individuals (two males) and the right well has paired individuals (a male and a female).

Copepods of the genus *Tigriopus*, which are common inhabitants of high intertidal rock pools, show contact mate-guarding behavior prior to copulation: in a guarding attempt, an adult *Tigriopus* male utilizes his geniculate antennae to capture a guarding target (juvenile or adult) [1, 8–10]. If the male succeeds in capture, he clasps the target with his first antennae on a dorsal hind edge of its cephalothorax (Fig 1A, S1 and S2 Movies) and initiates coordinated swimming with the target. Formed male-female guarding pairs are often maintained until copulation following the female's terminal molt, which can be up to two weeks in *T*. *californicus* [1].

One of the factors hypothesized to have selected for this mate-guarding trait in *Tigriopus* is its reproductive system: generally, an adult *Tigriopus* female mates with only one male in her lifetime [1] and produces up to several hundred offspring in multiple clutches after the copulation [11, 12]. In addition, a male-biased sex ratio, which is frequently cited as a possible factor contributing to prolonged mate guarding [13–18], is often observed in *Tigriopus* populations [19–21].

In spite of the male-skewed population attribute, *Tigriopus* has the ability to form mate-guarding pairs between a male and a female in a mass culture [10] with high accuracy (>95% in *T*. *californicus* [22]) and is expected to avoid male-male pair formation to secure reproductive outcome from the investment of long term of guarding. However, the mechanism allowing successful male-female pair formation is not yet well studied. Although previous work suggests that *Tigriopus* males have the ability to detect the presence of conspecific individuals and approach them [10, 20, 23], sex specificity of the approaching behavior has not been tested.

In this study, we analyzed behavior of both a capturer male and a target in mate-guarding attempts to explore phenomena underlying male-female pair formation in *T*. *californicus*. We first examined whether capturer males make guarding attempts selectively on females by comparing frequency and duration of capture between trials with a virgin female target and those with a virgin male target. Then, to investigate roles of a capturer and a target in successful male-female pairing, we examined whether those parameters change depending on excision of the antennae of a capturer and on physiological status of a target (alive or freshly-killed). We also tested whether capturer males make mate-guarding attempts more actively on a reproductively receptive (virgin) target than on an unreceptive (already mated) target to investigate if reproductive status of a target could affect interactions in formation of a mate-guarding pair.

## Materials and methods

### Animals

#### Rearing condition

Animals were reared in artificial seawater (salinity: 35 ppt) prepared by dissolving 43.6 g of Instant Ocean^®^ Sea Salt Mixture (Spectrum Brands, Inc., USA) in 1000 ml of distilled water and fed with powdered fish food (TetraVeggie^™^ eXtreme; Tetra Holding, Inc., USA) three times a week. Culture beakers and plates were maintained in incubators at 20°C with a 12-hour light:dark cycle. Evaporated rearing water was refilled with distilled water.

#### Population, collection and rearing

Original culture stocks were collected from high intertidal rock pools in San Diego, California, USA (32°45’N, 117°15’W), and maintained in 400-ml beakers for two months (equivalent to ~2 generations). The tested animals were obtained from gravid adult females randomly selected from the culture stocks. Each gravid female was kept in ~3 ml of artificial seawater in a well on a 6-well cell culture plate and removed from the well after it released an egg sac and the offspring started to hatch. The hatched nauplii were kept in the mass culture of siblings and collected as soon as they developed to copepodid stage I (CI), approximately 6 days after hatching. Each collected juvenile was individually maintained in a well on a 24-well cell culture plate until it developed to a given stage (CIII or adult). All individuals used in the behavioral tests were kept virgin until the tests except for the mated females used for an investigation of effect of reproductive status.

To evaluate accuracy of male-female pair formation, pairs with a juvenile from CII to CIV were collected from one of the rock pools described above (for the wild-caught group) and from one of the culture stocks (for the lab cultured group). Each pair was maintained in a well on a 24-well cell culture plate until the guarded juveniles developed to the adult stage.

#### Staging and sex identification

Developmental stages of animals were determined based on the number of exuviae molted in a rearing well and on morphology. A CIII juvenile was defined as a juvenile that had been collected at CI and molted two exuviae in its well. An adult male was defined as an individual that possessed clasping first antennae. An adult female was defined as an individual that possessed thin antennae and had molted five exuviae in its well since CI. Sex identification of CIII juveniles was performed after their sexual maturation (following the behavioral tests). These observations were performed under a stereoscopic microscope (Stemi SV 6, Carl Zeiss Microscopy, Switzerland).

#### Antennae excision and control surgery

Antennae excision and control surgeries were performed to investigate the role of the first antennae of males in mate guarding pair formation. In either the antennae excision or the control surgery group, each adult male was anesthetized with 3.5% MgCl_2_ in artificial seawater for 30 minutes and placed with its ventral side down on a paraffin sheet (Parafilm M^®^, Bemis Company Inc., USA) on a plastic Petri dish. The first antennae were spread out with tips of fine needles under a stereoscopic microscope. For the antennae excision surgery group, both of the first antennae were cut off at base with an ophthalmic standard incision scalpel with 15° blade edge angle (Micro Feather No. 715, Feather Safety Razor Co., Ltd., Japan). For the control surgery group, two legs (the 4th legs, which are comparable in length to the antennae) were excised with the same type of scalpel. After the surgeries, each of the adult males was individually placed in artificial seawater in a well of a 24-well cell culture plate and rested overnight for recovery.

#### Freeze-killing

Freeze-killed animals were prepared to analyze response of adult males to an immobile target. Prior to freeze-killing treatment, adults were individually placed in 400 μL of artificial seawater in a well on a 48-well cell culture plate (Tissue Culture Plate For Cell Attachment, TC treated, #748001; Nest Biotech Co., Ltd., China) on an LED light pad (Litup LED Light Pad Model LP4, Shenzhen Huion Animation Technology Co., Ltd., China) for 30 minutes. Then each adult was transferred into a 0.5 ml microtube on an aluminum cooling block precooled at -20°C in a freezer. The tubes on the block were immediately transferred back to the freezer and kept at -20°C for 10 minutes. Each frozen body was thawed in the tube with artificial seawater taken from the well in which the animal had been kept before freezing. The thawed bodies were returned to the wells and used for the behavioral test.

#### Preparation of mated females

To investigate effect of reproductive status of a female in mate-guarding pair formation, mated females were prepared by placing a virgin female and a virgin male together in a culture well and allowing them to mate in the well. Virgin animals were individually reared to the adult stage by the method described above and transferred into wells on 48-well cell culture plates containing 400 μL of artificial seawater with an individual of an opposite sex. A mated female was identified by an egg sac containing orange (i.e. fertilized) eggs at a posterior terminus of the urosome. To avoid possible effect of difference in apparent body size, each mated females was not used for the behavioral test until it released the egg sac attached to its body. After releasing the egg sac, each mated female was transferred to a new well and used for the behavioral test. Siblings of the mated females, which had been individually reared and kept virgin to the behavioral test, were used as”virgin” controls in the test.

### Behavioral tests

All the behavioral tests were performed in the artificial seawater at room temperature (approximately 23°C). Animals (capturers and targets) were fed at least one hour before a behavioral test. Each animal was collected with a Pasteur pipette from a rearing well on a culture plate and rinsed in artificial seawater in a Petri dish for several seconds to remove food debris. After that, it was transferred to a well on a 48-well cell culture plate containing 400 μL of artificial seawater on an LED light pad and kept in the well for 30 minutes for acclimation. After the acclimation (and the freeze-killing treatment when applicable), each target was transferred to a well of a capturer male with a Pasteur pipette (Fig 1A) and exposed to the capturer for 10 minutes.

### Video recording

A digital camera (EOS REBEL T6i, Canon, Japan) mounted above a test plate was used for recording and observation of behavior (Fig 1B). The video recording was started after the 30-minute acclimation and performed at 30 frames per second. The recording was stopped after the 10-minute observation period.

### Behavioral analysis

#### Duration of capture and frequency of guarding attempt

Initiation of guarding attempt by a capturer male was defined as a swift chase or a pounce that was immediately (in <0.5-s) followed by the contact of an antenna (for the males with their antennae surgically excised, the anterior end of the body) with any body part of the target. Duration of each time of capture was defined as the length of time from initiation of the attempt to a point when both antennae of a capturer male detached from the body of a target and was measured on the time scale of seconds. Frequency of guarding attempts was calculated as:

$$\frac{number\;of\;initiation\;events}{total\;observation\;period\;\left(min\right)-total\;duration\;of\;attempts\;\left(min\right)}$$

#### Statistic analysis

All statistical analyses were performed with Prism 7 for Mac OS X (Version 7.0a). Pairs that showed no guarding attempt were excluded from the analyses of capture duration.

## Results

### Sex-specific interruption of capture

To examine if males have a preference for the sex of capturing targets, we presented either a virgin adult male or a virgin adult female to individual males and video-recorded their guarding attempts. We calculated: 1) average duration of capture and 2) frequency of guarding attempts, and compared each index between male-female pairs and male-male pairs.

**Fig 2:**
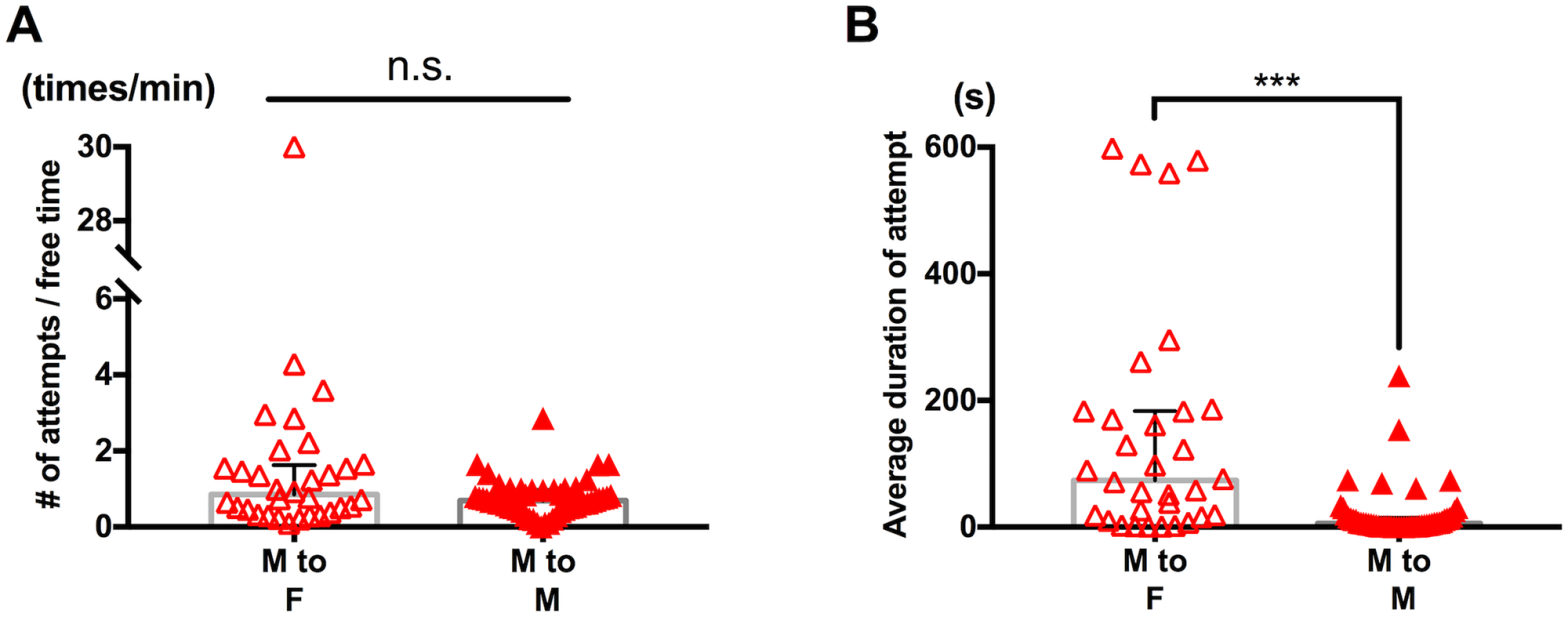
Difference in guarding attempts between male-female pairs and male-male pairs. Each triangle symbol represents data from one tested pair. Bars and whiskers represent medians and interquartile range (IQR) respectively. (A) Frequency of guarding attempts. Male to female (n = 32); male to male (n = 54). No significant difference was detected by Mann-Whitney *U* test (p = 0.13). (B) Average duration of capture was greater for male-female pairs (male to female (n = 32), male to male (n = 53). ***p<0.001 by Mann-Whitney *U* test).

While no significant difference was detected in the frequency of attempts between male-female pairs and male-male pairs (Fig 2A; p = 0.13, Mann-Whitney *U* test), a significant difference was detected in the average duration of capture (longer with females, Fig 2B; p<0.001, Mann-Whitney *U* test). These results suggest that males can be attracted to both males and females and sex-specific interruption or continuation of capture are involved in the process of mate-guarding pair formation.

### Reduction of attraction and sex-specific capture interruption in dead targets

In observations of intact male-male pairs, we noticed that the captured males were swimming vigorously as if trying to escape from the capturer (S3 and S4 Movies). Based on this observation, we formed a hypothesis that captured males take an active role in prevention of male-male pair formation by rejecting a capturer. To examine this hypothesis, we presented a dead body of either a freeze-killed male or female to individual males and analyzed response of the capturers.

**Fig 3:**
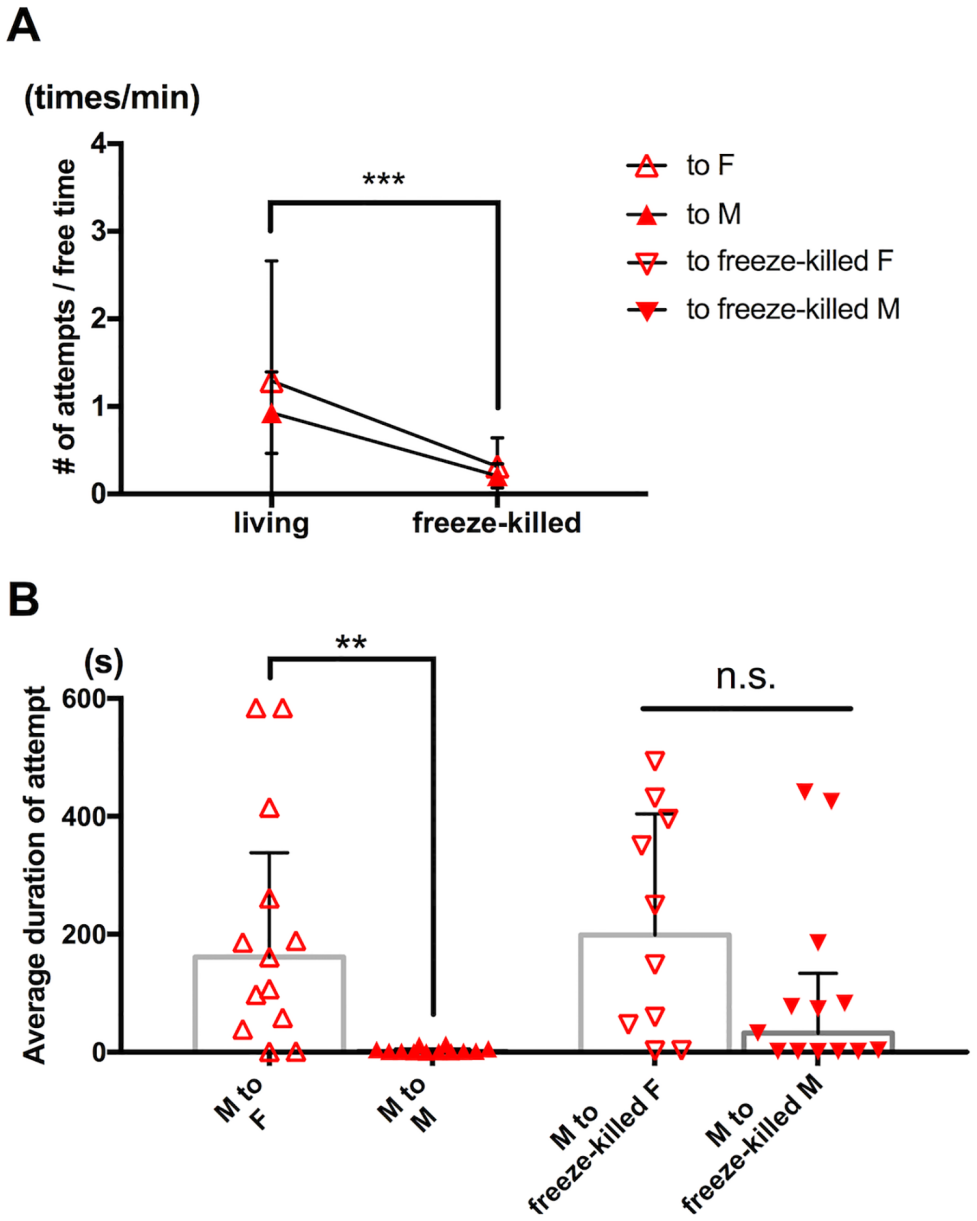
Reduction of attraction and sex-specific capture interruption in dead targets. (A) Frequency of guarding attempts. Male to living female (n = 13); male to living male (n = 12); male to freeze-killed female (n = 10); Male to freeze-killed male (n = 13). Triangle symbols and whiskers represent means and SD respectively. ***p<0.001 on treatment (freeze-killed or not) by two-way ANOVA. No significant effect of sex and no significant interaction between treatment and sex were detected (p = 0.24 and p = 0.52). (B) Average duration of capture. Male to living female (n = 13); male to living male (n = 12); male to freeze-killed female (n = 10); male to freeze-killed male (n = 13). Each triangle symbol represents data from one tested pair. Bars and whiskers represent medians and IQR respectively. **p<0.01 by Mann-Whitney *U* test. No significant difference was detected between male to freeze-killed female pairs and male to freeze-killed male pairs by Mann-Whitney *U* test (p = 0.097). No significant effect of sex and no significant interaction between treatment and sex were detected (p = 0.35 and p = 0.24).

The capturers showed significantly lower frequency of attempts on the dead body than on a living individual regardless of sex of the capturing targets (Fig 3A; p<0.001 on treatment, p = 0.24 on sex, p = 0.52 on interaction between treatment and sex; two-way ANOVA). In contrast to the sex-dependent difference in duration of capture of a living individual (Figs 2B and 3B; p<0.001 and p<0.01, Mann-Whitney *U* test), no significant difference in duration was detected depending on sex of a freeze-killed target (Fig 3B; p = 0.097, Mann-Whitney *U* test). While capture of the living males lasted less than 15 seconds (mean = 3.54, median = 1.155, maximum = 13.4; n = 12), the living females (mean = 206, median = 161.3, maximum = 584; n = 13), the freeze-killed females (mean = 217, median = 198.5, maximum = 493; n = 10), and the freeze killed males (mean = 91.0, median = 32.3, maximum = 441; n = 13) were held by the capturers for more than one minute on average (Fig 3B). These results suggest that an active rejection response by living males promotes interruption of male-male pairing. The reduced frequency of attempts (Fig 3A) also suggests that the supposed attractive signal was lost or inhibited in the freeze-killing treatment.

### Mating status-dependent rejection by females

Since females of *T*. *californicus* generally mate with only one male in their lifetime [1], mate-guarding pair formation between a male and a previously mated female is not reproductively beneficial for either sex. To investigate if either or both capturers and mated females avoid such pair formation, we presented either a virgin or mated adult female to individual males and compared duration of capture and frequency of attempts.

**Fig 4:**
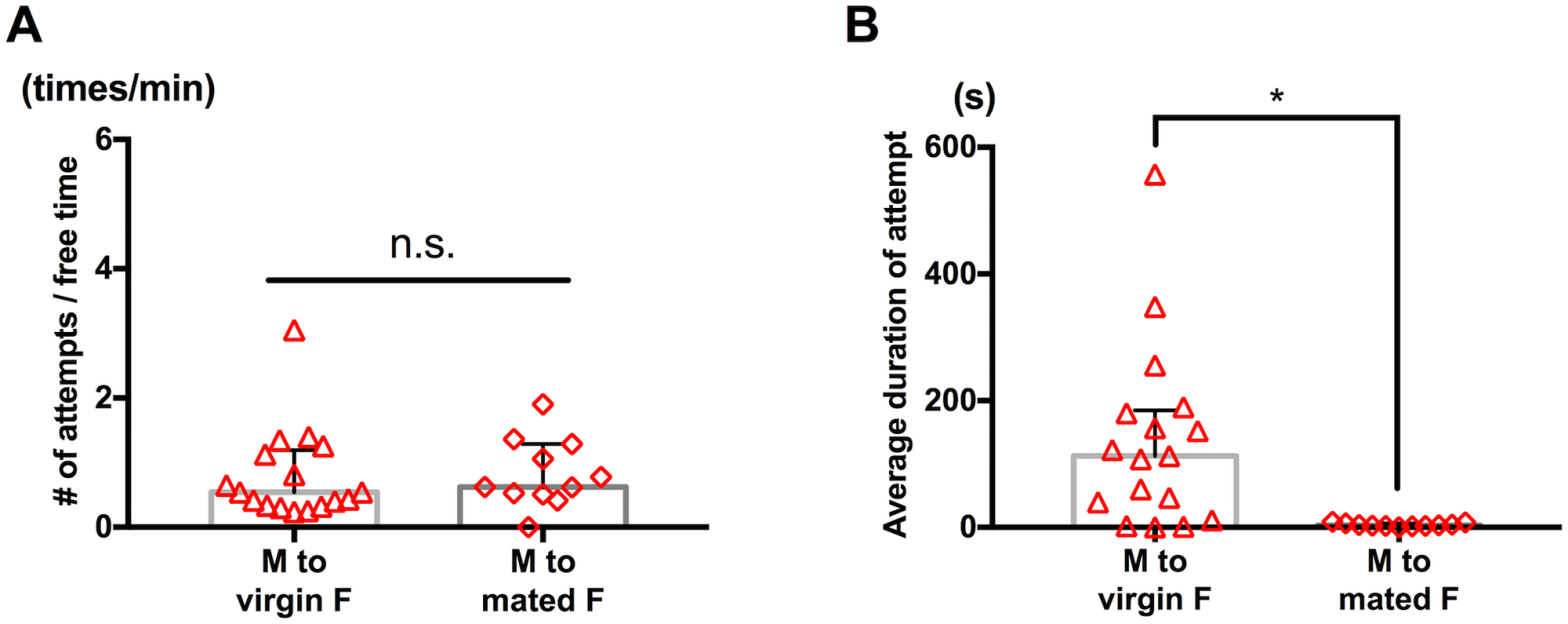
Difference in duration of capture depending on mating status of a target. Each symbol represents data from one tested pair. Bars and whiskers represent medians and IQR respectively. (A) Frequency of guarding attempts. Male to virgin female (n = 13); male to previously mated female (n = 9). No significant difference was detected by Mann-Whitney *U* test (p = 0.79). (B) Average duration of capture was greater for pairs with a virgin female target (male to virgin female (n = 13), male to previously mated female (n = 9). *p<0.05 by Mann-Whitney *U* test).

While no significant difference was detected in frequency of the attempts (Fig 4A; p = 0.79, Mann-Whitney *U* test), a significant difference was detected in the average duration of capture (longer with virgin females, Fig 4B; p<0.05, Mann-Whitney *U* test). These results suggest that while both virgin and mated females are attractive to males, mated females reject an attempt of pair formation.

### Higher attractiveness of adults compared to juveniles

Males of *Tigriopus* copepods are capable of clasping an adult female as well as an immature juvenile from copepodid stage II to V [1, 19, 24] (S2 Fig). However, since a female closer to sexual maturation requires a shorter guarding period before mating, it is hypothesized that a more developed female is a more favorable target for males in terms of reproductive benefit. Preference for a more developed female would potentially free a male to inseminate more females and thus to father a larger number of offspring in his lifetime.

**Fig 5:**
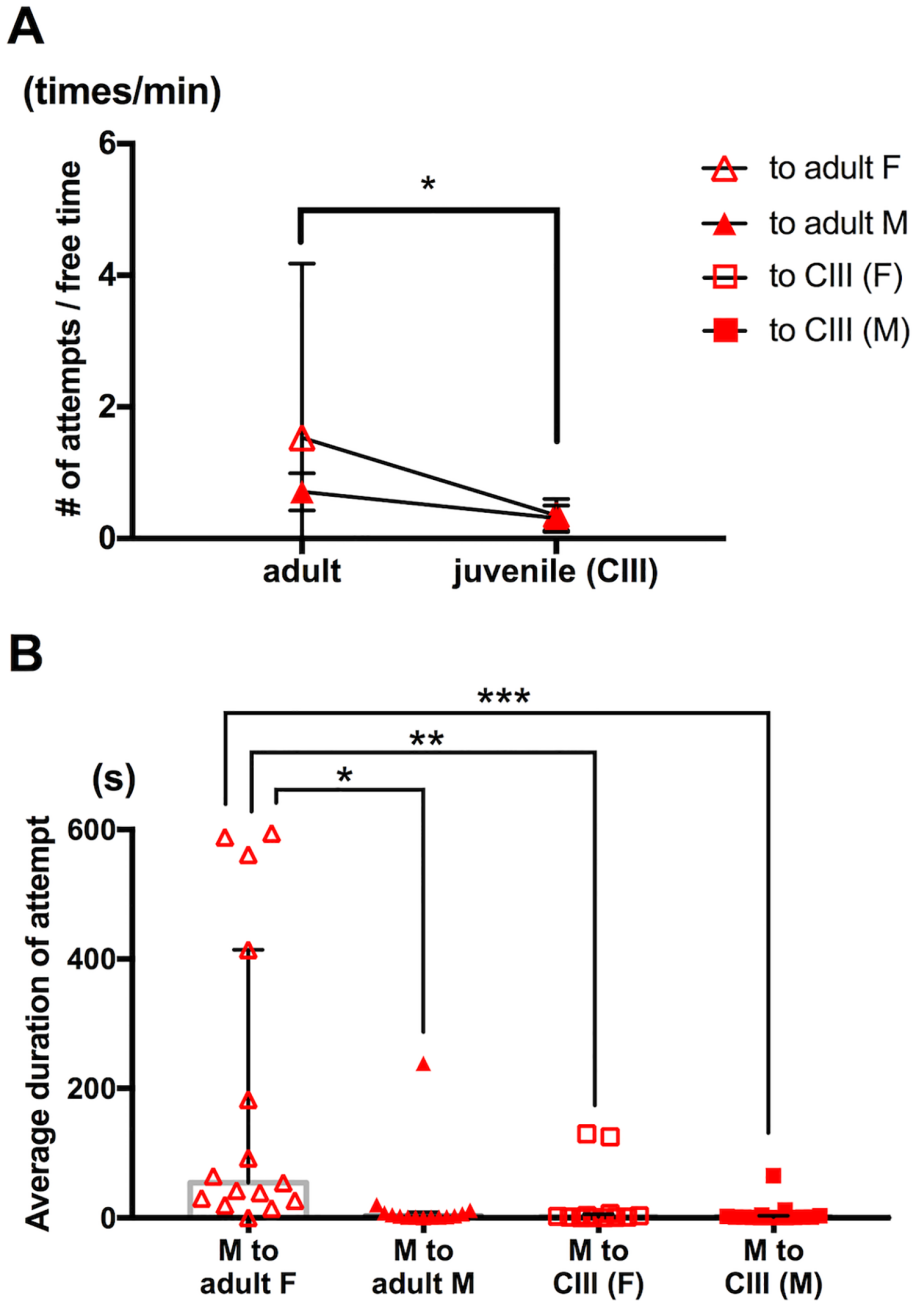
Higher attractiveness of adults compared to CIII juveniles. (A) Frequency of guarding attempts. Male to adult female (n = 15); male to adult male (n = 14); male to CIII female (n = 12); male to CIII male (n = 15). Symbols represent means; whiskers represent SD. *p<0.05 by two-way ANOVA. No significant effect of sex and no significant interaction between sex and stage were detected (p = 0.25 and p = 0.30). (B) Average duration of capture. Male to adult female (n = 15); male to adult male (n = 14); male to CIII female (n = 12); Male to CIII male (n = 15). Bars and whiskers represent medians and IQR respectively. *p<0.05, **p<0.01, ***p<0.001 by Kruskal-Wallis one-way ANOVA on ranks with pairwise multiple comparison according to Dunn's Method. No significant difference was detected between male to adult male, male to CIII female, and male to CIII male pairs (p>0.99 between male to adult male pairs and male to CIII female pairs, p = 0.69 between male to adult male pairs and male to CIII male pairs, p>0.99 between male to CIII female pairs and male to CIII male pairs).

To examine if a mature female is more attractive than a juvenile to males, we presented either an adult or a copepodid stage III (CIII) juvenile to individual males and examined if frequency of guarding attempts and average duration of capture changed depending on developmental stage of a target. The males showed significantly higher frequency of attempts on an adult than on a juvenile in general (Fig 5A; p<0.05 on developmental stage, p = 0.25 on sex, p = 0.30 on interaction between stage and sex; two-way ANOVA), demonstrating higher attractiveness of mature individuals compared to juveniles. The males clasped a virgin adult female for significantly longer time than a juvenile female, a juvenile male, and an adult male (Fig 5B; p<0.01, p<0.001, p<0.05; Kruskal-Wallis one-way ANOVA on ranks with pairwise multiple comparison according to Dunn's Method). No significant sex-dependent difference was detected between the two groups with a juvenile target (adult male to CIII female and adult male to CIII male) in both frequency of attempts and duration of capture (Fig 5; p = 0.91 and p = 0.88 in frequency, p>0.99 and p = 0.99 in duration; Kruskal-Wallis one-way ANOVA on ranks with pairwise multiple comparison according to Dunn's Method). Since morphological sexual dimorphism does not appear until the adult stage in *T*. *californicus*, we identified sex of the juvenile targets after their sexual maturation following the behavioral test.

### Significant role of antennae in approach to a target

The first antennae of adult male *Tigriopus* have a clasping hook and sensory bristles [25]. To examine if males use the first antennae to respond to presence of a target, we surgically removed them from males and observed guarding attempts of these males.

**Fig 6:**
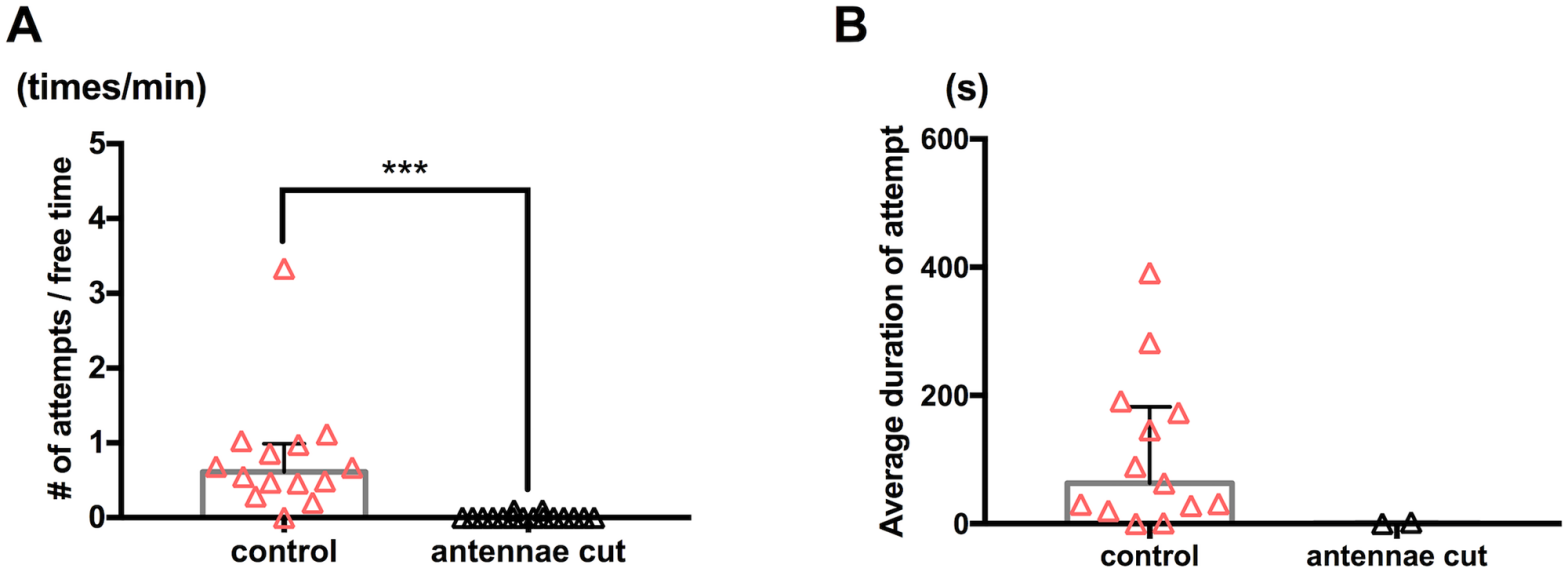
Role of antennae. Each triangle symbol represents data from one tested pair. Bars and whiskers represent medians and IQR respectively. (A) Frequency of guarding attempts. Control surgery male to intact female (n = 14); antennae cut male to intact female (n = 16). ***p<0.001 by Mann-Whitney *U* test. (B) Average duration of capture. Control surgery male to intact female (n = 13); antennae cut male to intact female (n = 2). Statistical analysis was not performed since there were only two individuals that showed a capturing attempt in the antennae cut group (n = 16, also see S1 Fig).

With their first antennae excised, males showed significantly lower frequency of attempts on a female compared to males in a control surgery (excision of two legs) group (Fig 6A; p<0.001, Mann-Whitney *U* test); fourteen out of sixteen males in the antennae cut group showed no attempt on a female, while thirteen out of fourteen males in the control surgery group showed one or more attempts during the observation period (S1 Fig). The two males that made a guarding attempt in the antenna cut group showed lower frequency of attempts (Fig 6A; 0.10 times/min) than any male that made attempts in the control surgery group (Fig 6A; minimum = 0.20 times/min, mean = 0.79 times/min, median = 0.61 times/min) and did not succeed in holding a female without their first antennae (Fig 6B). These results suggest an important role of the first antennae not only as clasping organs but also as detectors of a cue from a target in formation of a mate-guarding pair.

## Discussion

### Sex-specific rejection for male-female pair formation

Previous studies, including our own, supposed selective guarding attempts by capturer males to be critical in successful male-female pair formation in *Tigriopus* [1, 10, 23]. However, the present results suggest that it is captured individuals that play an active role in achievement of male-female pairing in adult *T*. *californicus*. While capturer males showed no significant difference in frequency of guarding attempts depending on sex of a target (Fig 2A), they terminated capture in a shorter time when their target was a living male (Fig 2B). This sex-dependent termination of capture was not observed in experiments with freshly killed targets (Fig 3B), suggesting importance of physiological condition of a target in interruption of capture. The vigorous movement observed in captured males (S3 Movie) might serve as a rejection signal and contributes to avoidance of male-male pair formation, eventually allowing both males involved an opportunity to form a pair with a female. Further analysis of swimming velocity and trajectory in guarding attempts would allow us to test whether movement of a captured individual is associated with termination of each time of capture by a capturer.

### Post-mating rejection in adult females

Capturer males terminated capture of a mated, reproductively unreceptive female in a shorter time compared to that of a virgin female (Fig 4B). This result suggests that mating and/or spawning experience alters the chemistry, morphology and/or behavior of adult females. In contrast to virgin females (S1 Movie), previously mated females showed vigorous movement when captured by a male (S5 Movie), which is a similar response to that of male targets in male-male pairs (S3 Movie).

Studies have reported post-mating alterations in female reproductive behavior in arthropods, especially in insects, suggesting that chemical and/or mechanical stimuli derived from males (e.g. seminal fluid peptides) induce the behavioral changes by activating particular neurons in females [26–29]. Given the mating system of *Tigriopus* in which females mate only once in their lifetime [1], such behavioral alternation would potentially help mated *Tigriopus* females to avoid cost of unnecessary paring. In future work, we would like to investigate the possibility of mating-induced behavioral modulation in *Tigriopus* females by quantitatively analyzing their behavioral response to a capturer. Gene expression analysis on females before and after mating as well as behavioral, genetic, and biochemical analyses on males may lead us to discover common ground in a system of post-mating behavioral change in females among arthropods.

### Attractive cues from targets

Some studies have suggested that diffusible chemicals derived from females serve as attractive cues for males in mate-guarding pair formation in *Tigriopus* species including *T*. *fulvus*, *T*. *japonicus*, *and T*. *californicus* [10, 20, 23]. However, sex specificity of the proposed cues was not examined in those studies. Our results support the existence of some attractive cue, but its sex specificity was not confirmed (Fig 2B). Considering the result of the experiment with freshly killed (i.e. immobile) targets (Fig 3A), we also expect movement of a target as a major, if not only, factor in attraction for males. Given the fact that *Tigriopus* is a dominant zooplankton in its habitats (tide pools not directly connected to ocean) and generally do not encounter other species, males of this species may be able to use a simple cue such as movement when searching for a mate. Active rejection by males and mated females along with other supplementary cues might complement a lack of specificity of a movement cue.

The result of the antennae excision test (Fig 6A) suggests that adult males utilize their first antennae to react to presence of a target. A neuroanatomical study on *T*. *californicus* showed sensory neurons in the first antennae to form two distinct axon tracts, a suggested motor sensory neuron tract and an olfactory receptor neuron tract, which are projecting to different regions in the brain [25]. Further behavioral analyses as well as molecular genetic and physiological analyses on these sensory neurons would allow us in future studies to reveal movement and/or substances that serve as the attractive cue.

The result of the behavioral test with juvenile and adult targets (Fig 6B) suggests that a cue from a target is capable of carrying information on developmental stage of the target by either a qualitative or a quantitative means. This is consistent with previous studies with *T*. *californicus* and *T*. *japonicus* in which capturer males showed a tendency to switch their guarding target from a juvenile to a more mature female in the same container [1, 23]. When multiple females are available, *Tigriopus* males might utilize information on developmental stage of the targets to assess a potential guarding period required before mating, thereby maximizing cost-efficiency of mate-guarding behavior. We expect a search for the attractive cue to also clarify if the information is transmitted by the attractive substances suggested by previous studies [20, 23] or other stimuli.

### Model for male-female pair formation between adults

**Fig 7:**
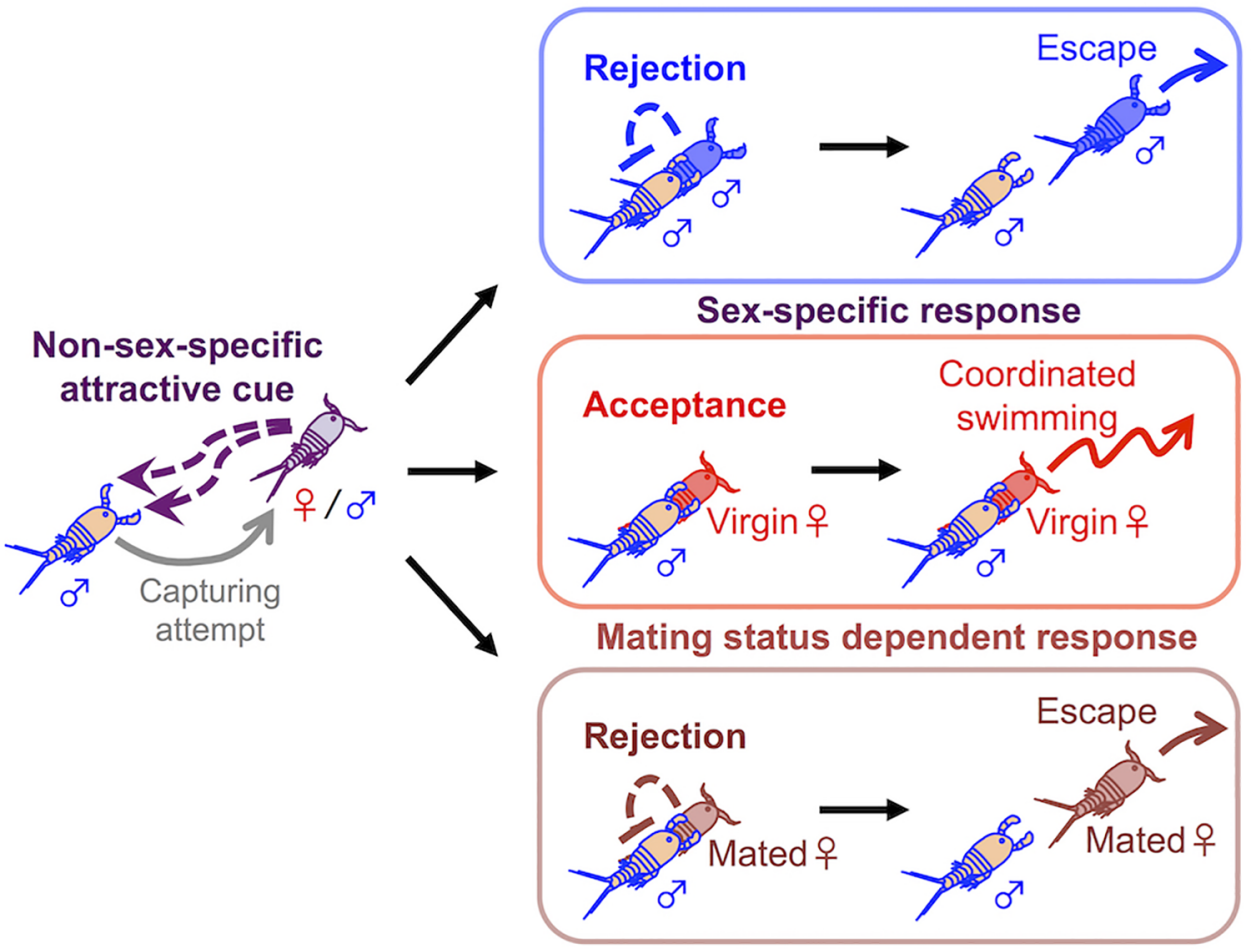
Behavioral model: Interaction between a capturer and a target in pair formation. Shaded individuals are targets and orange individuals are capturer males.

Considering the present results, we propose a model of interactions between a capturer and a target in mate guarding pair formation of adult *T*. *californicus* (Fig 7). In this model, a capturer male is attracted by a non-sex-specific cue derived from a target and initiates a guarding attempt on the target (Fig 7, left); when the target is a male, the capturer receives a rejection response from the male target and releases him (Fig 7: top); when the target is a virgin female, the capturer can be accepted by the female target and forms a pair with her (Fig 7: middle-middle and middle-right); in a case that the target is a female but she has already mated, the capturer is rejected by the unreceptive target and releases her (Fig 7: bottom). This model proposes a mechanism in which a target takes an active role to allow a reproductive success from the mate-guarding behavior between adults in *Tigriopus*.

Although *T*. *californicus* shows high accuracy in male-female pair formation [22], it is reported that males of *T*. *japonicus*, a related species of *T*. *californicus*, often form and keep pairs with male targets [24]. Behavioral, molecular, and/or genetic comparison between these species may help us to understand the mechanism allowing successful male-female pair formation in *Tigriopus*.

### Pair formation with juvenile targets

In the experiment with juvenile targets, we did not find a significant difference in either frequency of attempts or duration of capture depending on sex of the targets (Fig 5). However, despite a male-biased sex ratio in populations [9, 21], adult *T*. *californicus* males form male-female pairs with juvenile targets with high accuracy both in natural and laboratory conditions. For example, in our observation on pairs collected from a tide pool and a laboratory culture stock, we found all of guarded juveniles that survived to the adult stage developed into females (S1 Table). Also, in a large-scale crossing experiment performed in our laboratory, there was only one juvenile that developed into a male among more than 1,000 juveniles collected from formed pairs (Thiago G Lima, personal communication). These male-male pairing rates in adult-juvenile pairs are comparable to or even smaller than those reported in adult pairs (0.6–3.5% [22]).

One possible explanation for the eventual male-female pairing in adult-juvenile pairs is that a successful pair formation between an adult male and a juvenile target occurs through a longer period of interaction. To investigate this possibility, we would need to observe their behavior for an extended time in future studies. Another possibility is that sexual differentiation of juveniles is altered by capturer males. Sex determination in *Tigriopus* appears to be polygenic [13, 30] and may also be influenced by external factors, including salinity, presence of chemical compounds, pressure, temperature, and pH [19, 21, 31, 32]. Further studies on physical and chemical communications between adult males and guarded juveniles may provide a clue to the unclear sex determination system in *Tigriopus* as well as evidence of a previously unknown benefit of mate-guarding behavior for capturers (differentiation of targets into females).

## Supporting information

S1 Movie Male-to-female guarding attempt (played at actual speed). The capturer (adult male) approaches the target (adult female) and captures the target with his antennae. S2 Movie shows the same event in slow motion.
(MP4)

S2 Movie Male-to-female guarding attempt (slow motion). The same event as S1 Movie is played at 2x slower speed. The capturer (adult male) approaches the target (adult female) and catches a caudal part of the target with his antennae (00:08). Then the capturer crawls along the dorsal part of the target in a rostral direction and reaches up a dorsal hind edge of cephalothorax of the target (00:12).
(MP4)

S3 Movie Male-to-male guarding attempt (played at actual speed). The capturer (adult male) approaches the target (adult male) and catches him, but the capturer is detached from the target in 7 seconds after the first contact. S4 Movie shows the same event in slow motion.
(MP4)

S4 Movie Male-to-male guarding attempt (slow motion). The same event as S3 Movie is played at 2x slower speed. The capturer (adult male) approaches the target (adult male) and catches the target with his antennae. The capturer holds a caudal part of the target (00:08) and starts to crawl along the dorsal part of the target whilst the target vigorously swims around. Finally, the capturer is detached from the target (00:22).
(MP4)

S5 Movie Male-to-mated female guarding attempt (played at actual speed). The capturer (adult male) approaches the target (mated adult female) and catches her, but the capturer is detached from the target in 6 seconds after the first contact. S6 Movie shows the same event in slow motion.
(MP4)

S6 Movie Male-to-mated fmale guarding attempt (slow motion). The same event as S5 Movie is played at 2x slower speed. The capturer (adult male) approaches the target (mated adult female) and catches the target with his antennae. The capturer holds a caudal part of the target (00:06) and crawls along the dorsal part of the target whilst the target vigorously swims around. Finally, the capturer is detached from the target (00:18).
(MP4)

S1 Fig Decreased guarding attempts in antennae cut males. Total number of guarding attempts. Each triangle symbol represents data from one tested pair. Bars and whiskers represent medians and IQR respectively. Control surgery male to intact female (n = 14); antennae cut male to intact female (n = 16). **p<0.01 by Mann-Whitney *U* test.
(TIFF)

S2 Fig Developmental stages of *T*. *californicus* females. *T*. *californicus* undergo six nauplius stages (from NI to NV), five copepodid stages (from CI to CV) and an adult stage [33]. Males are capable of clasping juveniles from CII to CV stages and adult females.
(TIFF)

S1 Table Sex of guarded juveniles. Wild-caught (n = 24): pairs collected from the field collection site. Lab cultured (n = 24): pairs collected from the lab culture stocks. Pairs with a juvenile from CII to CIV were collected from the sources and maintained separately from other pairs in wells of 24-well plates until the guarded juveniles developed to the adult stage.
(XLSX)

S1 Alternative Language Abstract Japanese abstract provided by Satomi Tsuboko-Ishii. (PDF)

## References

[1] Burton RS. Mating System of the Intertidal Copepod *Tigriopus californicus*. Marine Biology. 1985;86(3):247–52. doi: 10.1007/Bf00397511

[2] Hockham LR, Vahed K. The function of mate guarding in a field cricket (Orthoptera: Gryllidae;*Teleogryllus natalensis* otte and cade). Journal of Insect Behavior. 1997;10(2):247–56. doi: 10.1007/bf02765557

[3] Yokoi S, Okuyama T, Kamei Y, Naruse K, Taniguchi Y, Ansai S, et al An essential role of the arginine vasotocin system in mate-guarding behaviors in triadic relationships of medaka fish (*Oryzias latipes*). PLoS Genet. 2015;11(2):e1005009 doi: 10.1371/journal.pgen.1005009 ; 25719383 10.1371/journal.pgen.1005009 PMC4342251

[4] Getz LL, Carter CS, Gavish L. The Mating System of the Prairie Vole, *Microtus ochrogaster*—Field and Laboratory Evidence for Pair-Bonding. Behavioral Ecology and Sociobiology. 1981;8(3):189–94. doi: 10.1007/Bf00299829

[5] Setchell JM, Charpentier M, Wickings EJ. Mate guarding and paternity in mandrills: factors influencing alpha male monopoly. Animal Behaviour. 2005;70:1105–20. doi: 10.1016/j.anbehav.2005.02.021

[6] Buss DM. Human mate guarding. Neuro Endocrinol Lett. 2002;23 (Suppl. 4):23–9. . 12496732

[7] Edward DA, Stockley P, Hosken DJ. Sexual Conflict and Sperm Competition. Cold Spring Harbor Perspectives in Biology. 2015;7(4). doi: 10.1101/cshperspect.a017707 25301931 10.1101/cshperspect.a017707 PMC4382747

[8] Fraser JH. The Occurrence, Ecology and Life History of *Tigriopus fulvus* (Fischer). Journal of the Marine Biological Association of the United Kingdom. 1936;20(03):523–36.

[9] Alexander HJ, Richardson JM, Anholt BR. Multigenerational response to artificial selection for biased clutch sex ratios in *Tigriopus californicus* populations. J Evol Biol. 2014;27(9):1921–9. doi: 10.1111/jeb.12449 . 25039835 10.1111/jeb.12449

[10] Lazzaretto I, Salvato B., Libertini A. Evidence of Chemichal Signaling in *Tigriopus fulvus* (copepoda, harpacticoida). Crustaceana. 1990;59(2):171–9.

[11] Vittor BA. Effects of the environment on fitness-related life history characters in *Tigriopus californicus*: The University of Oregon; 1971.

[12] Hagiwara A, Lee C. -S., Shiraishi D. J. Some reproductive characteristics of the broods of the harpacticoid copepod *Tigriopus japonicus* cultured in different salinities. Fisheries Science. 1995;61(4):618–22.

[13] Alexander HJ, Richardson JM, Edmands S, Anholt BR. Sex without sex chromosomes: genetic architecture of multiple loci independently segregating to determine sex ratios in the copepod *Tigriopus californicus*. J Evol Biol. 2015;28(12):2196–207. doi: 10.1111/jeb.12743 . 26332493 10.1111/jeb.12743

[14] Parker GA. Courtship Persistence and Female-Guarding as Male Time Investment Strategies. Behaviour. 1974;48(1):157–84. doi: 10.1163/156853974x00327

[15] Grafen A, Ridley M. A Model of Mate Guarding. Journal of Theoretical Biology. 1983;102(4):549–67. doi: 10.1016/0022-5193(83)90390-9

[16] Jormalainen V. Precopulatory mate guarding in crustaceans: Male competitive strategy and intersexual conflict. Quarterly Review of Biology. 1998;73(3):275–304. doi: 10.1086/420306

[17] Rondeau A, Sainte-Marie B. Variable mate-guarding time and sperm allocation by male snow crabs (*Chionoecetes opilio*) in response to sexual competition, and their impact on the mating success of females. Biological Bulletin. 2001;201(2):204–17. doi: 10.2307/1543335 11687392 10.2307/1543335

[18] Coxworth JE, Kim PS, McQueen JS, Hawkes K. Grandmothering life histories and human pair bonding. Proc Natl Acad Sci U S A. 2015;112(38):11806–11. doi: 10.1073/pnas.1599993112 ; 26351687 10.1073/pnas.1599993112 PMC4586877

[19] Egloff DA. Ecological aspects of sex ratio and reproduction in experimental and field populations of the marine copepod *Tigriopus californicus*. Palo Alto, California: Stanford University; 1966.

[20] Lazzaretto I, Franco F, Battaglia B. Reproductive-Behavior in the Harpacticoid Copepod *Tigriopus fulvus*. Hydrobiologia. 1994;293:229–34.

[21] Voordouw MJ, Anholt BR. Environmental sex determination in a splash pool copepod. Biological Journal of the Linnean Society. 2002;76(4):511–20. doi: 10.1046/j.1095-8312.2002.00087.x

[22] Peterson DL, Kubow KB, Connolly MJ, Kaplan LR, Wetkowski MM, Leong W, et al Reproductive and phylogenetic divergence of tidepool copepod populations across a narrow geographical boundary in Baja California. Journal of Biogeography. 2013;40(9):1664–75. doi: 10.1111/jbi.12107

[23] Kelly LS, Snell TW, Lonsdale DJ. Chemical communication during mating of the harpacticoid *Tigriopus japonicus*. Philosophical Transactions of the Royal Society B-Biological Sciences. 1998;353(1369):737–44. doi: 10.1098/rstb.1998.0239

[24] Ito T. Taxonomy within the genus Tigriopus (Copepoda: Harpacticoida) from Japan, with reference to the relationship between *Tigriopus japonicus* and *T*. *californicus*. Annual report of the Seto Marine Biological Laboratory. 1988;2:28–35. (Japanese)

[25] Andrew DR, Brown SM, Strausfeld NJ. The minute brain of the copepod *Tigriopus californicus* supports a complex ancestral ground pattern of the tetraconate cerebral nervous systems. J Comp Neurol. 2012;520(15):3446–70. doi: 10.1002/cne.23099 . 22431149 10.1002/cne.23099

[26] Avila FW, Sirot LK, LaFlamme BA, Rubinstein CD, Wolfner MF. Insect seminal fluid proteins: identification and function. Annu Rev Entomol. 2011;56:21–40. doi: 10.1146/annurev-ento-120709-144823 ; 20868282 10.1146/annurev-ento-120709-144823 PMC3925971

[27] Yang CH, Rumpf S, Xiang Y, Gordon MD, Song W, Jan LY, et al Control of the postmating behavioral switch in Drosophila females by internal sensory neurons. Neuron. 2009;61(4):519–26. doi: 10.1016/j.neuron.2008.12.021 ; 19249273 10.1016/j.neuron.2008.12.021 PMC2748846

[28] Yapici N, Kim YJ, Ribeiro C, Dickson BJ. A receptor that mediates the post-mating switch in *Drosophila* reproductive behaviour. Nature. 2008;451(7174):33–U1. doi: 10.1038/nature06483 18066048 10.1038/nature06483

[29] Obara Y, Fukano Y, Watanabe K, Ozawa G, Sasaki K. Serotonin-induced mate rejection in the female cabbage butterfly, *Pieris rapae crucivora*. Naturwissenschaften. 2011;98(11):989–93. doi: 10.1007/s00114-011-0847-3 . 21947195 10.1007/s00114-011-0847-3

[30] Ar-Rushdi AH, editor The polygenic basis of sex-ratio in *Tigriopus* Xth International Congress of Genetics; 1958; McGill University, Montreal, Canada.

[31] Vacquier VD, Belser WL. Sex Conversion Induced by Hydrostatic Pressure in the Marine Copepod *Tigriopus californicus*. Science. 1965;150(3703):1619–21. doi: 10.1126/science.150.3703.1619 . 17743973 10.1126/science.150.3703.1619

[32] Egami N. A Note on the Sex-differentiaon of the Marine Copepod, *Tigriopus japonicus*. Anotationes Zoologicae Japonenses. 1951;24(3):131–6.

[33] Raisuddin S, Kwok KW, Leung KM, Schlenk D, Lee JS. The copepod *Tigriopus*: a promising marine model organism for ecotoxicology and environmental genomics. Aquat Toxicol. 2007;83(3):161–73. doi: 10.1016/j.aquatox.2007.04.005 . 17560667 10.1016/j.aquatox.2007.04.005

