# Supplementary figures and images for: Sex-specific rejection in mate-guarding pair formation in the intertidal copepod, Tigriopus californicus
Source: PLoS One. 2017 Aug 23;12(8):e0183758. doi: 10.1371/journal.pone.0183758 (PMC5568411; doi:10.1371/journal.pone.0183758)

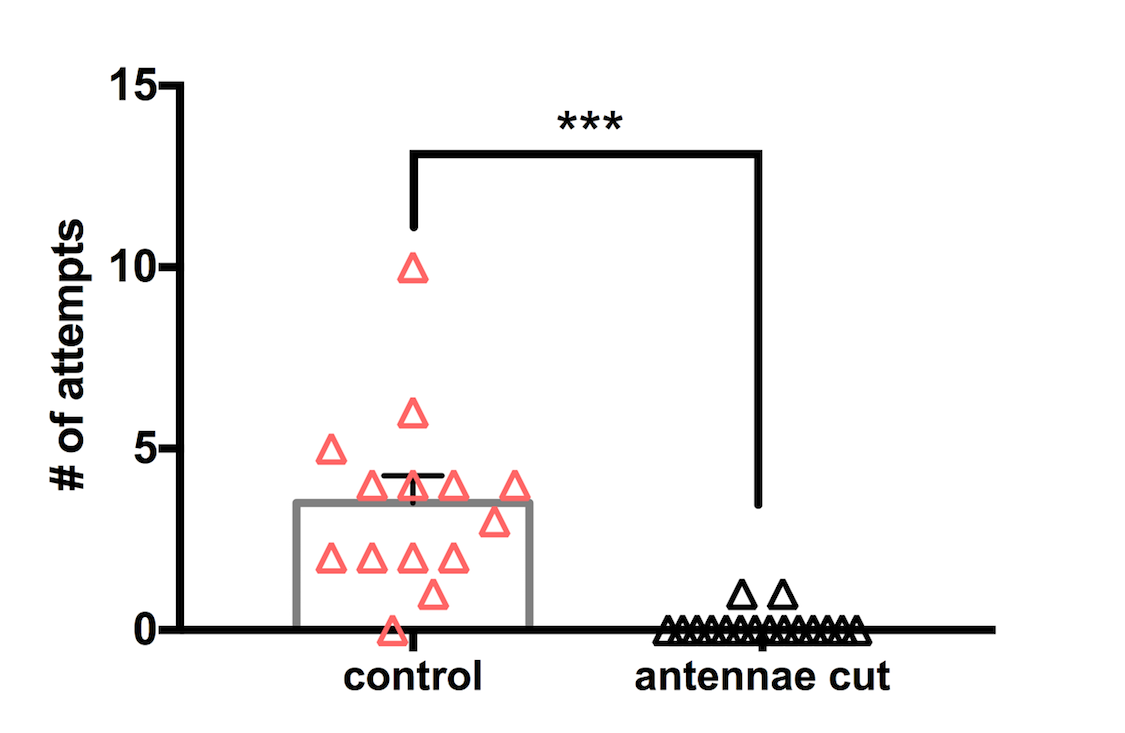

Supplement: S1 Fig — Total number of guarding attempts. Each triangle symbol represents data from one tested pair. Bars and whiskers represent medians and IQR respectively. Control surgery male to intact female (n = 14); antennae cut male to intact female (n = 16). **p<0.01 by Mann-Whitney U test. (TIFF) [file pone.0183758.s007.tiff]

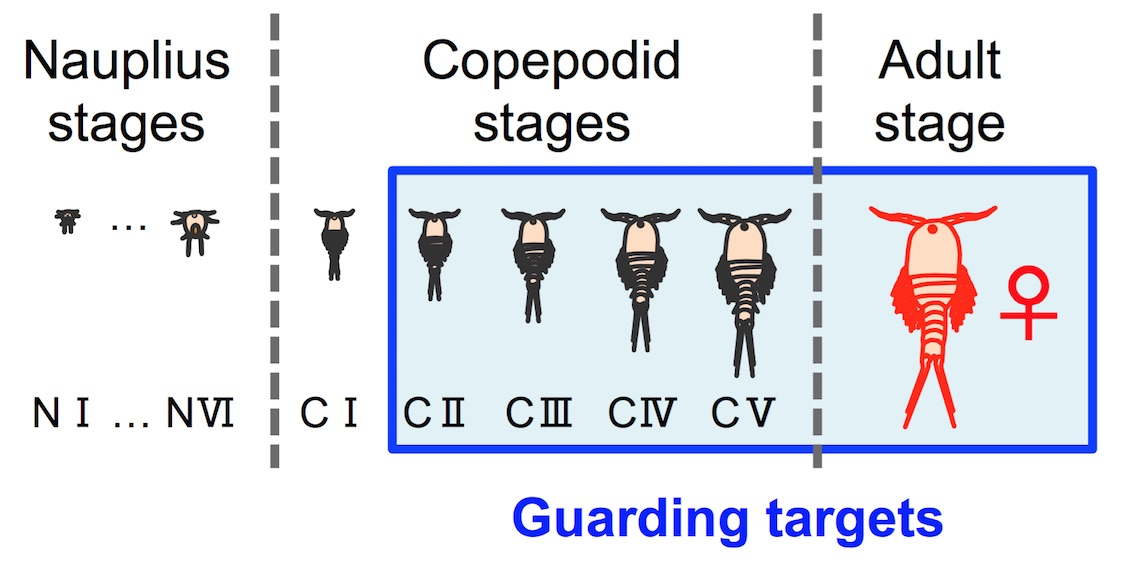

Supplement: S2 Fig — T. californicus undergo six nauplius stages (from NI to NV), five copepodid stages (from CI to CV) and an adult stage [33]. Males are capable of clasping juveniles from CII to CV stages and adult females. (TIFF) [file pone.0183758.s008.tiff]
