# Supplementary material for: Sex-specific rejection in mate-guarding pair formation in the intertidal copepod, Tigriopus californicus
Source: PLoS One. 2017 Aug 23;12(8):e0183758. doi: 10.1371/journal.pone.0183758 (PMC5568411; doi:10.1371/journal.pone.0183758)
Supplement: S1 Alternative Language Abstract — (PDF) [file pone.0183758.s010.pdf]

交配相手の確保は、動物の有性生殖において最も重要な過程の一つである。潮間帯性のカイアシ類（copepods）であるシオダマリミジンコ（*Tigriopus*）属は、オスがメスを捕捉し、交配までの最大 2 週間メスを把持し続けるという配偶者防衛行動（mate-guarding behavior）を示す。シオダマリミジンコ属はオス-メス間での防衛ペア形成を高い精度で行うが、ペア形成時に両性の間で行われる相互作用は充分に記載されておらず、オス-メス間でのペア形成を成功させる機構は未解明である。

本研究で、我々はカリフォルニアシオダマリミジンコ（*Tigriopus californicus*）を用いた実験を行い、防衛ペア形成時の捕捉者（オス）と捕捉対象（メスまたはオス）双方の行動を分析した。捕捉者オスはどちらの性別の個体にも誘引された一方で、捕捉対象がオスであった場合の捕捉行動は、対象がメスであった場合の捕捉行動よりも有意に短い時間で中断された。しかし、捕捉者オスにメスまたはオスの死体を提示した場合には、その死体の性別を問わず、生きたメスを捕捉対象とした試行と同程度の時間、捕捉者オスは死体の把持を続けた。

我々の結果は、捕捉されたオスによって発せられる性特異的な拒否シグナルが、オスどうしでのペア形成を妨げていることを示唆している。また、実験からは、交配経験のあるメスがペア形成の試みを拒否することも示唆された。我々の知る限り、本研究は、シオダマリミジンコ属において、捕捉された個体による積極的な拒否が、生殖の成功につながるオス-メス間防衛ペア形成の促進に関与していることを示唆する初めての研究である。
